# Social and physical neighbourhood characteristics and loneliness among older adults: results from the MINDMAP project

**DOI:** 10.1136/jech-2020-214217

**Published:** 2020-11-05

**Authors:** Erik Timmermans, Irina Motoc, J Mark Noordzij, Marielle A Beenackers, Rita Wissa, Aliou Sarr, Asli Gurer, Guillaume Fabre, Milagros Ruiz, Dany Doiron, Joost Oude Groeniger, Dorly Deeg, Frank J Van Lenthe, Martijn Huisman

**Affiliations:** 1 Department of Epidemiology and Biostatistics, Amsterdam UMC - Locatie VUMC, Amsterdam, The Netherlands; 2 Department of Public Health, Erasmus University Medical Center, Rotterdam, The Netherlands; 3 Research Institute of the McGill University Health Centre, Montreal, Canada; 4 Research Department of Epidemiology and Public Health, University College London, London, United Kingdom; 5 Department of Public Administration and Sociology, Erasmus University, Rotterdam, The Netherlands; 6 Department of Human Geography and Spatial Planning, Utrecht University, Utrecht, The Netherlands; 7 Department of Sociology, Vrije Universiteit Amsterdam, Amsterdam, The Netherlands

**Keywords:** Environmental health, Epidemiology of ageing, Neighbourhood/place, Mental health

## Abstract

**Background:**

Loneliness is associated with several adverse mental and physical health outcomes in older adults. Previous studies have shown that a variety of individual-level and perceived area-level characteristics are associated with loneliness. This study examined the associations of objectively measured social and physical neighbourhood characteristics with loneliness.

**Methods:**

We used cross-sectional data from 1959 older adults (63–98 years) who participated in the Longitudinal Ageing Study Amsterdam (LASA; wave 2011/12) and the Health and Living Conditions of the Population of Eindhoven and Surroundings study (GLOBE; wave 2014) in the Netherlands. Study-specific loneliness scores were harmonised across both cohort studies and divided into tertiles denoting low, medium and high levels of loneliness. Objectively measured neighbourhood characteristics, including area-level percentages of low educated residents, social security beneficiaries and unoccupied dwellings, average income, crime levels and land use mix, were linked to individual-level data. Multinomial logistic regression analyses were conducted to examine the associations of interest.

**Results:**

There was no statistical evidence for an association of the included neighbourhood characteristics with loneliness. Although not statistically significant, the observed associations suggested that participants living in neighbourhoods with more heterogeneous land use mix were less likely to have a medium and high level of loneliness than those living in more homogeneous neighbourhoods in terms of land use mix (OR_medium_=0.54, 95% CI=0.18–1.67; OR_high_=0.67, 95% CI=0.21–2.11).

**Conclusion:**

The results indicate that the included objectively measured social and physical neighbourhood characteristics are not associated with loneliness in old age.

## INTRODUCTION

Loneliness can be considered as the outcome of the subjective and negative evaluation of the gap between an individual’s desired and actual quantity and quality of social relationships.^1^ In particular, older adults are considered to be vulnerable to loneliness, because they are highly susceptible to age-related changes, such as the loss of one’s partner and friends through death or deteriorating health, and loss of social roles through retirement.^2^ The prevalence of loneliness among older adults is substantial across European countries, including the Netherlands (eg, 28.5% in 64–84-years-olds and 50.0% in 85–94-years-olds in 2011/12).^3 4^ Loneliness is considered a growing public health problem, particularly among older adults in industrialised countries.^5^ Because loneliness is associated with several adverse mental (eg, depression) and physical (eg, mortality) health outcomes in old age, identifying determinants of loneliness in older adults should be considered a public health priority.^2 6^

Although a large body of evidence on individual-level determinants of loneliness, such as low socioeconomic position and poor functional status, exists, only a few recent studies have focused on the associations between environmental characteristics and loneliness in older adults.^2^ Social relationships are embedded within and shaped by a wider social structural context that includes neighbourhood characteristics.^7 8^ To the extent that characteristics of a neighbourhood influence the social ties that form between its residents, this may have implications for the extent to which they feel lonely.^8 9^ Previous studies show, for instance, that lower levels of perceived neighbourhood walkability, safety and attachment are associated with a higher level of loneliness among older adults.^10–14^ Research on the association between objectively measured neighbourhood characteristics and loneliness in older adults is, however, lacking. The results of such studies may provide additional guidance for urban planners and policymakers in designing more appropriate and contextualised strategies for interventions to prevent or reduce loneliness.

A variety of objectively measured social and physical neighbourhood characteristics, such as socioeconomic conditions, crime and land use mix, has been suggested to affect loneliness in old age. However, empirical evidence on the strength and direction of these associations is lacking. Older adults living in socioeconomically deprived areas, characterised by lower area-levels of education and income and higher percentages of social security beneficiaries and unoccupied dwellings, may be faced with lower levels of neighbourhood quality and other environmental challenges, such as nuisance, in their local living environment. This may make them less likely to participate in the community and to invest in social relationships.^2 15–17^ On the other hand, the homogeneous composition of socioeconomically deprived neighbourhoods (eg, in terms of educational level) may enhance social cohesion and interactions among residents, which may protect against loneliness.^18^ Neighbourhood crime may undermine older adults’ feelings of trust and may lead them to feel alienated from other people.^8^ Neighbourhood crime is therefore expected to be positively associated with loneliness in old age. Access to neighbourhood resources, such as supermarkets, sport facilities and parks, has been shown to be important for older adults in performing activities to meet daily needs, and contributes to initiating and maintaining social interactions with community members.^19^ Indeed, people who use more resources report less loneliness.^11^ Higher levels of land use mix, reflecting the availability of various destinations and neighbourhood resources in the local living environment, are therefore expected to be associated with lower levels of loneliness among older adults.^20^

This study extends previous research by examining initial cross-sectional associations of objectively measured social and physical neighbourhood characteristics, including area-level percentages of low educated residents, social security beneficiaries and unoccupied dwellings, average income, crime levels, and land use mix, with loneliness in older adults, independent of individual-level characteristics.

## METHODS

### Study design and sample

We used data from older individuals enrolled in the Longitudinal Ageing Study Amsterdam (LASA) and the Health and Living Conditions of the Population of Eindhoven and Surroundings (GLOBE) study, two Dutch cohorts participating in the MINDMAP (Promoting mental well-being and healthy ageing in cities) project. The MINDMAP project, LASA and GLOBE have been described in detail previously.^21–24^ In short, the MINDMAP project aims to identify the opportunities and challenges posed by urban environmental characteristics for the promotion and management of mental well-being and cognitive functioning of older individuals, by bringing together longitudinal studies from various countries, enriched with area-level environmental exposures and social and urban policy indicators.^21^ For this study, LASA (wave 2011/12) and GLOBE (wave 2014) were selected, because harmonised data on loneliness and the environment were available in the MINDMAP project for these waves in these two Dutch cohort studies. To our best knowledge, nothing major changed in terms of loneliness interventions/policies during the 2-year period between the LASA and GLOBE wave. The LASA study was started in 1992, and is an ongoing longitudinal cohort study in the Netherlands that studies the determinants, trajectories and consequences of physical, cognitive, emotional and social functioning in older adults.^22 23^ The GLOBE study was initiated in 1991, and is an ongoing longitudinal cohort study that has been designed to assess mechanisms and factors explaining socioeconomic inequalities in health in the Netherlands.^24^

LASA wave 2011/12 and GLOBE wave 2014 included 1522 and 4851 respondents, respectively. A total of 1308 LASA-respondents (63–102 years) who participated in the main face-to-face interview were included in the MINDMAP project.^25^ Because this study focuses on older adults and the LASA respondents were at least 63 years old at wave 2011/12, 2059 GLOBE respondents aged 63 years and older were eligible for inclusion in this study. Of all 3367 eligible individuals, 1408 (41.8%) individuals were excluded due to missing data on loneliness (n=110), on at least one neighbourhood characteristic (n=918) and on at least one confounder (n=380). As a result, the analysed sample consisted of 1959 individuals with complete data. The included and excluded group did not significantly differ in loneliness, neighbourhood income and crime. On average, area-level percentages of low-educated residents and unoccupied dwellings and land use mix were higher in the neighbourhoods of the excluded group, while area-level percentage of social security beneficiaries was higher in the neighbourhoods of the included group. Together, these data do not suggest underrepresentation of, for example, relatively deprived groups. The MINDMAP project, LASA and GLOBE were approved by the Ethical Review Boards of their respected institutions and are conformed to the principles embodied in the Declaration of Helsinki.

### Dependent variable

#### Loneliness

In LASA and GLOBE, loneliness was measured using the 11-item and six-item version of the De Jong Gierveld Loneliness Scale, respectively.^26 27^ The study-specific scale score was harmonised using tertiles and dummy-coded (0=no, 1=yes) into: low level of loneliness (reference category), medium level of loneliness, and high level of loneliness.

In LASA, the scale score ranged from 11 to 33 and tertiles were defined as low (score=11), medium (score=12–14), and high (score=15–33). Scores were calculated for respondents with data on at least nine items. If one or two items were missing, scores on the completed items were summed and divided by the number of items completed and multiplied by 11. If respondents missed three or more items, scores were not calculated and coded as missing.

In GLOBE, the scale score ranged from 6 to 30 and tertiles were defined as low (score=6–9), medium (score=10–12), and high (13–30). Scores were calculated for respondents with data on at least five items. If one item was missing, scores on the completed items were summed and divided by five, then multiplied by six. If respondents missed two or more items, scores were not calculated and coded as missing.

### Independent variables

#### Social neighbourhood characteristics

Nationwide data on social neighbourhood characteristics were retrieved from Statistics Netherlands.^28–32^ In the Netherlands, six-digit postal code areas (average area size: 0.0025 km^2^), four-digit postal code areas (average area size: 8.3 km^2^) and neighbourhoods (average area size: 3.1 km^2^) are geographically delineated areas and include, on average, approximately 15, 1870 and 630 households, respectively.^33^ Data on average income, percentage of social security beneficiaries, and percentage of unoccupied dwellings were retrieved at the neighbourhood-level. As described elsewhere, Geographic Information System software (ArcGIS version 10.1; ESRI) was used to link these neighbourhood data to individual-level cohort data using residential six-digit postal codes of respondents.^33^ Data on the percentage of social security beneficiaries and crime were also retrieved at the neighbourhood-level, and were linked to individual-level cohort data using neighbourhood codes of respondents. The area-level data on educational level were retrieved at the four-digit postal code area-level and were linked to individual-level cohort data using residential four-digit postal codes of respondents. The area-level data on income and crime were collected for the year 2011. The area-level data on social security benefits, unoccupied dwellings and educational level were collected for the years 2010, 2012 and 2014, respectively.

*Educational level* was measured as the percentage of residents in four-digit postal code areas who attained a low educational level (ie, ≤ lower secondary education).^28^ *Average income* was measured as the rounded average gross year income in €1000,- of residents in the neighbourhood.^29^ *Percentage of social security beneficiaries* was measured as the percentage of residents in the neighbourhood receiving general social assistance according to the Social Assistance Act and Work and Social Assistance Act.^30^ *Percentage of unoccupied dwellings* was calculated by dividing the number of unoccupied dwellings in the neighbourhood by the total neighbourhood housing stock, and multiplied by 100.^31^ *Neighbourhood crime* was measured as the number of criminal offences (ie, property crimes, destructions, and violent crimes) per 1000 residents in the neighbourhood.^32^

#### Physical neighbourhood characteristic

*Land use mix* was calculated in 1000-m buffer zones around the respondent’s home address using the following entropy formula:

$$\text{Land use mix score=}-\text{1*}\left[\sum_{\text{k}}\left({\text{p}}_{\text{k}}\text{*ln}\left({\text{pk}}_{\text{k}}\right)\right)\right]\text{/ln}\left(\text{N}\right),$$

in which p is the proportion of land area within the 1000-m buffer zone devoted to a specific land use and N is the total number of land use categories within the 1000-m buffer zone, which was 10 in this study.^20 34^ The area size of an 1000-m buffer zone is 3.1 km^2^. This area size corresponds with the average area size of a Dutch neighbourhood.^33^

For the assessment of the land use mix score, the proportions of the following 10 land use categories within the 1000-m buffer zone were assessed using Urban Atlas 2012 data in QGIS Desktop (version 2.18.20; QGIS Development Team): built-up areas, industrial and commercial areas, infrastructure, ports, urban green areas, sports and leisure facilities, agricultural land, other natural areas, blue spaces, and other areas.^35^ The land use mix score represents a measure of heterogeneity and ranges from 0 to 1, with 0 representing no land use mix and 1 representing a perfect mix of land use categories.^20 34^

### Individual-level confounders

*Age* in years, *sex* (0=man, 1=woman), *study* (0=LASA, 1=GLOBE), partner status, educational level and household income were included as individual-level confounders in the analyses.

*Partner status* of respondents was assessed by asking whether they were married or living with a spouse or a partner in a common household (0=no partner, 1=partner). *Educational level* was assessed as the highest level of education completed by the respondent in accordance with the International Standard Classification of Education (0=upper secondary education or lower, 1=post-secondary non-tertiary education or higher).^36^ *Household income* was measured by indicating whether the net annual household income was above or below the net mean household income (in 2012: €25 100.-; in 2014: €29 000,-) in the Netherlands (0=no, 1=yes).^37^

### Area-level confounder

*Population density* was included as area-level confounder in the analyses. Population density was measured as the number of residents per square kilometre in the neighbourhood and categorised into quartiles, with higher quartiles representing higher population density.^28^

### Statistical analysis

Characteristics of the study sample and the neighbourhoods were presented using descriptive statistics. Pearson correlations were assessed between all neighbourhood characteristics.

Pooled multinomial logistic regression analyses were conducted to examine the association between each neighbourhood characteristic and loneliness, separately. All associations were examined in models constructed step by step. In Model 1, the associations were adjusted for age, sex and study. In Model 2, the associations were additionally adjusted for all other individual-level characteristics (ie, partner status, educational level and household income). In Model 3, the associations were additionally adjusted for area-level population density. All statistical analyses were performed in RStudio (version 3.6.0; R Foundation).

## RESULTS

The mean age of all 1959 (n_LASA_=417 and n_GLOBE_=1542) participants was 72.8 (SD=6.7) years with an age-range of 63–98 years (table 1). Just over half of the participants were men (51.0%). Of all participants, 614 (31.3%), 632 (32.3%) and 713 (36.4%) reported a low, medium and high level of loneliness, respectively. The majority of GLOBE participants (69.9%) had a household income below the country-specific net mean household income. This percentage was 47.2% in LASA.

**Table 1:** Characteristics of the pooled sample and of the LASA (wave 2011/12) and GLOBE (wave 2014) sample

Characteristics	Pooled (n=1959)	LASA (n=417)	GLOBE (n=1542)
**Age in years (mean±SD)**	72.8±6.7	72.5±7.0	72.9±6.6
**Sex (%)**			
Men	51.0	50.4	51.2
Women	49.0	51.6	48.8
**Partner status (%)**			
No partner	28.2	30.0	27.8
Partner	71.8	70.0	72.2
**Educational level (%)**			
Upper secondary education or lower	73.8	78.2	72.6
Post-secondary non tertiary education or higher	26.2	21.8	27.4
**Household income (%)**			
Below country-specific net mean household income	65.1	47.2	69.9
Above country-specific net mean household income	34.9	52.8	30.1
**Loneliness (%)**			
Low level of loneliness	31.3	43.6	28.0
Medium level of loneliness	32.3	33.3	32.0
High level of loneliness	36.4	23.0	40.0
**Study (%)**			
LASA	21.3	100.0	0.0
GLOBE	78.7	0.0	100.0
**Area-level population density (%)**			
Quartile 1 (10–2679 residents per square kilometre)	25.4	21.3	26.5
Quartile 2 (2680–4293 residents per square kilometre)	24.8	22.1	25.5
Quartile 3 (4294–5725 residents per square kilometre)	25.0	32.6	23.0
Quartile 4 (5726–25 681 residents per square kilometre)	24.8	24.0	25.0

### Neighbourhood characteristics

On average, the percentages of low educated residents, social security beneficiaries and unoccupied dwellings in the neighbourhoods where participants were living were 43.4% (SD=7.1%), 1.8% (SD=1.6%), and 4.2% (SD=3.2%), respectively (table 2). The mean average income in these areas was 22.5 k€ (SD=4.8 k€). The average number of criminal offences per 1000 residents in these neighbourhoods was 63.1 (SD=84.5). The average land use mix score was 0.73 (SD=0.11). The correlations between neighbourhood characteristics were weak to moderate (table 3).

**Table 2:** Objectively measured social and physical neighbourhood characteristics

Characteristics	Mean±SD	Range
Percentage of low educated residents	43.4±7.1	19.0–62.0
Average income in €1000,-	22.5±4.8	15.0–63.0
Percentage of social security beneficiaries	1.8±1.6	0.0–9.0
Percentage of unoccupied dwellings	4.2±3.2	0.0–26.0
Number of criminal offences per 1000 residents	63.1±84.5	5.0–1584.0
Land use mix score	0.73±0.11	0.15–1.00

**Table 3:** Pearson correlations between neighbourhood characteristics*

Neighbourhood characteristics	1	2	3	4	5	6	7
1. Population density†	1.00						
2. Percentage of low educated residents	−0.11	1.00					
3. Average income in €1000,-	−0.33	−0.51	1.00				
4. Percentage of social security beneficiaries	0.50	0.23	**−0.57**	1.00			
5. Percentage of unoccupied dwellings	−0.06	−0.37	0.26	−0.09	1.00		
6. Number of criminal offences per 1000 residents	0.10	−0.20	0.10	0.10	0.37	1.00	
7. Land use mix score	−0.11	0.04	0.10	−0.02	−0.11	−0.03	1.00

*In bold: p<0.05.

†The continuous measure of population density was used in these analyses.

### Neighbourhood characteristics and loneliness

Results from the multinomial regression analyses are presented in table 4. The age-, sex- and study-adjusted model indicated that participants who lived in neighbourhoods with relatively many low-educated residents were more likely to have a medium and high level of loneliness than a low level of loneliness (Model 1: OR_medium_=1.02, 95% CI=1.00–1.03; OR_high_=1.02, 95% CI=1.00–1.03). After additional adjustment for all individual-level confounders (Model 2: OR_medium_=1.01, 95% CI=1.00–1.03; OR_high_=1.01, 95% CI=0.99–1.03) and population density (Model 3: OR_medium_=1.01, 95% CI=1.00–1.03; OR_high_=1.01, 95% CI=0.99–1.03), these associations were attenuated.

**Table 4:** Associations between neighbourhood characteristics and loneliness in the study sample†,‡

	Model 1§		Model 2¶		Model 3**	
	Medium level of loneliness OR (95% CI)	High level of loneliness OR (95% CI)	Medium level of loneliness OR (95% CI)	High level of loneliness OR (95% CI)	Medium level of loneliness OR (95% CI)	High level of loneliness OR (95% CI)
**Social neighbourhood characteristics**						
Percentage of low educated residents	**1.02 (1.00–1.03)**	**1.02 (1.00–1.03)**	1.01 (1.00–1.03)	1.01 (0.99–1.03)	1.01 (1.00–1.03)	1.01 (0.99–1.03)
Average income in €1000,-	0.98 (0.96–1.00)	**0.96 (0.94–0.98)**	0.99 (0.96–1.01)	0.98 (0.96–1.00)	0.98 (0.95–1.00)	0.98 (0.96–1.01)
Percentage of social security beneficiaries	1.04 (0.96–1.12)	**1.12 (1.04–1.21)**	1.01 (0.94–1.10)	1.06 (0.98–1.14)	1.06 (0.97–1.16)	1.06 (0.97–1.16)
Percentage of unoccupied dwellings	0.97 (0.94–1.01)	0.98 (0.95–1.02)	0.97 (0.94–1.01)	0.98 (0.95–1.02)	0.96 (0.93–1.00)	0.99 (0.95–1.02)
Number of criminal offences per 1000 residents	1.00 (1.00–1.00)	1.00 (1.00–1.00)	1.00 (1.00–1.00)	1.00 (1.00–1.00)	1.00 (1.00–1.00)	1.00 (1.00–1.00)
**Physical neighbourhood characteristic**						
Land use mix	0.59 (0.20–1.75)	0.64 (0.22–1.89)	0.59 (0.20–1.76)	0.78 (0.26–2.36)	0.54 (0.18–1.67)	0.67 (0.21–2.11)

†The reference category of the outcome measure is low level of loneliness.

‡In bold: p<0.05.

§In Model 1, the associations are adjusted for the individual-level confounders age, sex and study.

¶In Model 2, the associations are additionally adjusted for the individual-level confounders partner status, educational level and household income.

**In Model 3, the associations are additionally adjusted for the area-level confounder population density.

Participants who lived in neighbourhoods with a high average income were less likely to have a high level of loneliness than a low level of loneliness (Model 1: OR_high_=0.96, 95% CI=0.94–0.98). After additional adjustment for all individual-level confounders (Model 2: OR_high_=0.98, 95% CI=0.96–1.00) and population density (Model 3: OR_high_=0.98, 95% CI=0.96–1.01), this association was attenuated.

Participants who lived in neighbourhoods with relatively many social security beneficiaries were more likely to have a high level of loneliness than a low level of loneliness (Model 1: OR_high_=1.12, 95% CI=1.04–1.21). After additional adjustment for all individual-level confounders (Model 2: OR_high_=1.06, 95% CI=0.98–1.14) and population density (Model 3: OR_high_=1.06, 95% CI=0.97–1.16), this association was attenuated.

Although not statistically significant, the age-, sex- and study-adjusted associations suggested that participants who lived in areas with higher levels of land use mix were less likely to have a medium and high level of loneliness than a low level of loneliness (Model 1: OR_medium_=0.59, 95% CI=0.20–1.75; OR_high_=0.64, 95% CI=0.22–1.89). This was also found after additional adjustment for all individual-level confounders (Model 2: OR_medium_=0.59, 95% CI=0.20–1.76; OR_high_=0.78, 95% CI=0.26–2.36) and population density (Model 3: OR_medium_=0.54, 95% CI=0.18–1.67; OR_high_=0.67, 95% CI=0.21–2.11).

No statistical evidence was found for an association of area-level percentage of unoccupied dwellings and neighbourhood crime with loneliness (Models 1–3).

## DISCUSSION

This study aimed to examine the associations between objectively measured social and physical neighbourhood characteristics and loneliness in older adults. No statistical evidence was found for an association of objectively measured social neighbourhood characteristics, including area-level percentages of low educated residents, social security beneficiaries and unoccupied dwellings, average income, and crime levels, with loneliness in old age. Although not statistically significant, the observed associations suggest that older adults living in neighbourhoods with more heterogeneous land use mix were less likely to have a medium and high level of loneliness than those living in more homogeneous neighbourhoods in terms of land use mix.

Compared with previous studies, which have mainly focused on individual-level and perceived area-level determinants of loneliness, an innovative aspect of this study is the focus on objectively measured social and physical environmental characteristics in relation to loneliness in older adults. An additional strength of this study is the use of data from the MINDMAP data platform that allowed us to include harmonised data from two large-scale population-based cohort studies in the Netherlands, which have increased the statistical power of the analyses.^21^

There are several caveats. First, the social-environmental data that were used in this study are related to administrative areas.^28–32^ These assessments might not exactly characterise the spaces within which participants actually move and therefore might create spatial uncertainty relating to actual exposure, as well as not accounting for within- and between-person heterogeneity in spatial habits.^38^ This may have led to an underestimation of the associations and may have concealed associations between social neighbourhood characteristics and loneliness. If the social-environmental data would have been examined in personalised exposure areas, as has been done for land use mix in 1000-m buffer zones around participant’s home addresses, we might have found stronger associations between the social neighbourhood characteristics and loneliness. Another caveat concerns temporal mismatches between area-level data and cohort data. However, it is not likely that these mismatches have led to an underestimation of associations in the present study, because the included area-level data are considered to be stable over a time window of 4 years.^39^

The results show that the social neighbourhood characteristics included in our analyses are not associated with loneliness in older adults, after adjusting for individual-level socioeconomic and household indicators, and area-level population density. In addition to methodological issues related to exposure,^38^ a more substantial explanation for our null findings could be that, in particular, perceptions of social neighbourhood conditions are more important determinants of loneliness or behaviour and feelings that affect loneliness, such as social participation and feelings of trust, safety, social cohesion and neighbourhood attachment, than objective neighbourhood conditions of which individuals may not always be aware of.^40^

Previous research found that older adults who use more neighbourhood resources are less lonely.^11^ The findings of this study suggest that older adults living in neighbourhoods with higher levels of land use mix, which means that various resources and destinations are available in their local living environment, are less likely to have stronger feelings of loneliness. Access to neighbourhood resources invites people to use them and to participate in the community and to initiate and maintain social interactions among community members, which may protect against loneliness.^19^ Although the association of land use mix with loneliness was not statistically significant, it might be worthwhile to investigate this association in future studies and to examine the pathways between land use mix and loneliness.

This cross-sectional study shows that the included social and physical neighbourhood characteristics are not associated with loneliness in old age. Studies with a longitudinal design are needed to assess whether changes in environmental characteristics are actually associated with changes in loneliness in old age. By combining Geographic Information System data and Global Positioning System data, future studies could assess the actual activity space and environmental exposure of participants, which could help to better examine the actual impact of environmental characteristics on loneliness in older adults. Furthermore, to obtain a more comprehensive understanding of these associations, future research could examine how objective and perceived neighbourhood characteristics interact and determine loneliness in old age. Additionally, to examine whether the findings of this study are generalisable to other contexts, future research could be conducted in neighbourhoods across different countries and cultures.

**What is already known on this subject**

- The prevalence of loneliness in old age is substantial, and loneliness is associated with several adverse mental and physical health outcomes in older adults. A variety of individual-level and perceived area-level characteristics are associated with loneliness.

**What this study adds**

- The present study extends the current literature by examining associations of objectively measured social and physical neighbourhood characteristics with loneliness in older adults. This study did not find statistical evidence for an association of objectively measured social neighbourhood characteristics, including area-level percentages of low educated residents, social security beneficiaries and unoccupied dwellings, average income, and crime levels, with loneliness in old age. Although not statistically significant, the observed associations suggest that older adults living in neighbourhoods with higher levels of land use mix, which means that various resources and destinations are available in their local living environment, are less likely to have stronger feelings of loneliness.
